# Supplementary material for: Safety of Oral Carica papaya L. Leaf 10% Ethanolic Extract for Acute and Chronic Toxicity Tests in Sprague Dawley Rats
Source: Toxics. 2024 Mar 1;12(3):198. doi: 10.3390/toxics12030198 (PMC10975813; doi:10.3390/toxics12030198)
Supplement: Supplementary file 1 [file toxics-12-00198-s001.zip › toxics-supplementary-data.pdf]

toxics

MDPI

Supplementary Data

Safety of Oral *Carica papaya* L. Ethanolic Extract from Acute and Chronic Toxicity Tests in Sprague-Dawley Rats

Table S1. Pesticide residue examination results.

**Organochlorines group**	**Results (mg/kg)**	**Organochlorines group**	**Results (mg/kg)**
α-BHC	not found	Endosulfan I	not found
γ-BHC (Lindane)	not found	Dieldrin	not found
β-BHC	not found	Endrin	not found
δ-BHC	not found	4,4′-DDD	not found
Heptaclor	not found	Endosulfan II	not found
Aldrin	not found	4,4′-DDT	not found
Heptaclor epoxide (Isomer B)	not found	Endrin aldehyde	not found
Trans-Chlordane	not found	Methoxychor	not found
Cis-Chlordane	not found	Endosulfan sulfate	not found
4-4′-DDE	not found	Endrin ketone	not found
**Organophosphates group**	**Results (mg/kg)**	**Organophosphates group**	**Results (mg/kg)**
Diazinon	not found	Chlopyrifos	not found
Parathion-methyl	not found	Pirimiphos-ethyl	not found
Fenitrothion	not found	Profenofos	not found
Malathion	not found	Ethion	not found
**Pyrethroids group**	**Results (mg/kg)**	**Pyrethroids group**	**Results (mg/kg)**
Lamda-cyhalothrin	not found	Cypemethrin	not found
Pemethrin	not found	Fenvarelat	not found
Cyfluthrin	not found	Deltamethrin	not found
**Carbamates group**	**Results (mg/kg)**	**Carbamates group**	**Results (mg/kg)**
Aldicarb	not found	Oxamyl	not found
Aldicarb sulfine	not found	Pirimicarb	not found
Carbaryl	not found	Promecarb	not found
Carbafuran	not found	Ethimfos	not found
Methiocarb	not found		

Note. Limit of Detection (LOD) = 0.01 mg/kg

Table S2. Types and contents of phenolic and flavonoid compounds in the analysis of production in each batch of papaya leaf extract.

	Quantity detected (mg/ 100 g dry extract)			
**Compounds**	**Batch No.1**	**Batch No.2**	**Batch No.3**	**Batch No.4**
Gallic acid	873.5 ± 8.5	845.7 ± 7.3	921.5 ± 8.2	895.6 ± 7.5
Rutin	1043 ± 7.2	978.6 ± 6.9	1105 ± 7.4	1082 ± 6.5
Catechin	421.5 ± 5.8	413.2 ± 4.6	409.8 ± 5.2	453.6 ± 4.9
Rosmarinic acid	nd	nd	nd	nd
Luteolin	nd	nd	nd	nd
Quercetin	853.7 ± 6.4	812.4 ± 5.7	887.6 ± 6.2	864.3 ± 5.2
Apigenin	105.6 ± 4.8	87.47 ± 3.5	119.6 ± 4.9	110.3 ± 3.7
Kaempferol	18.56 ± 1.7	17.03 ± 1.5	20.64 ± 2.2	19.58 ± 1.8

	Quantity detected (mg/ 100 g dry extract)		
**Compounds**	**Batch No.5**	**Batch No.6**	**Batch No.7**
Gallic acid	882.6 ± 7.5	915.6 ± 7.4	878.4 ± 6.8
Rutin	1024 ± 8.7	1087 ± 9.6	1051 ± 7.4
Catechin	418.3 ± 5. 4	427.2 ± 6.6	420.6 ± 5.5
Rosmarinic acid	nd	nd	nd
Luteolin	nd	nd	nd
Quercetin	846.1 ± 6.3	865.4 ± 7.0	852.9 ± 6.0
Apigenin	107.3 ± 3.8	112.6 ± 4.3	102.3 ± 3.5
Kaempferol	21.52 ± 1.6	22.78 ± 1.9	19.83 ± 1.7

Note. nd: not detectable

Disclaimer/Publisher's Note: The statements, opinions and data contained in all publications are solely those of the individual author(s) and contributor(s) and not of MDPI and/or the editor(s). MDPI and/or the editor(s) disclaim responsibility for any injury to people or property resulting from any ideas, methods, instructions or products referred to in the content.
